# Preface of the Special Issue: “Peritoneal Surface Malignancies (PSM): The SICO (Italian Society of Surgical Oncology) PSM—Oncoteam Experience, Result Analysis, and Studies’ Purpose”

**DOI:** 10.3390/cancers13246387

**Published:** 2021-12-20

**Authors:** Paolo Sammartino, Marco Vaira

**Affiliations:** 1 Cytoreductive Surgery and HIPEC Unit, Pietro Valdoni Department of Surgery, Policlinico Umberto I, Sapienza University of Rome, 00161 Rome, Italy; 2 Surgical Oncology Unit, Candiolo Cancer Institute, FPO-IRCCS, 10060 Candiolo, Italy; marco.vaira@ircc.it

Over the past 40 years, strategies to treat neoplastic spread into the peritoneal space have benefitted from a gradually evolving approach, thanks mainly to studies conducted by the charismatic leader in this medical field Professor Paul Sugarbaker, Washington DC. Despite the international oncological community’s initial skepticism, and thanks to other pioneers who followed his example in some European countries, especially France (Francois Gilly and Dominique Elias) and Holland (Frans Zoetmulder), the concept has over time gained ground that peritoneal neoplastic spread must in many conditions be regarded as a locoregional disease amenable also to locoregional treatment. The principal characteristics distinguishing this branch of oncological surgery, right from the beginning and on Paul Sugarbaker’s intuition, were to standardize a specific procedure for surgical cytoreduction (peritonectomy procedures) and to combine this technique with perioperative intraperitoneal chemotherapy (eventually combined with hyperthermia) conceived as a locoregional procedure to complete the surgical demolition [1]. Over time, a voluminous literature accumulated on the numerous series for each single pathology characterized in its clinical progression by metastatic spread into the peritoneum, ideally still today represented by the term peritoneal surface malignancies (PSM). The specific problems related to every neoplasia underlying peritoneal spread result in widely differing indications, therapeutic strategies, and outcomes [2]. These differences explain why the disease fascinates us and puts it among the partly unexplored frontiers in surgical oncology.

Oncological experts today concentrate their attention on three aspects. The first is the real incidence of the results obtained with perioperative chemotherapy usually combined with surgical cytoreduction. On this subject, contrasting data have been published according to the various primary tumors responsible for peritoneal spread and the ulterior variable, namely, the various drugs used for intraperitoneal therapy make the topic even more complex and still unclear [3].

The second problem this surgical oncology chapter raises is the increasingly apparent need for a multidisciplinary approach. Apart from the evident need for coordinating medical oncology staffing so as to integrate systemic and locoregional chemotherapy, numerous other specialists must contribute to correctly manage these patient’s disease. The critical aspects regarding diagnostics, anesthesia, and reanimation, the pathologic examination of surgical specimens, the biomolecular research analysis able to supply prognostic variables and last, rehabilitation, nutritional and psychologic problems mean that in managing patients with PSM numerous dedicated specialists are indispensable.

Lastly, experience common in recent years to all the major referral centers for treating PSM shows that even though patients referred to them have increased in number, owing to advances in selection criteria and the need to treat patients having the best prognostic factors, this increase has left the number of procedures conducted with curative intent unchanged [4]. This consideration converges ideally with all the studies so far undertaken, especially in colorectal and gastric cancer, to verify whether in primary locally advanced tumors, the first surgical intervention could include measures to prevent peritoneal spread, a development that in these patients is often too extensive to allow treatment with curative intent. Although the available data remain controversial, this research direction could, in future, for certain neoplastic sites, change the surgical approach in numerous situations [5].

In our country, Italy, for some years now, the Italian Society of Oncologic Surgery, has established a specific Oncoteam comprising the referral centers for treating PSM and as in other European countries, centralizes patient data and promotes clinical studies. This Special Issue intends to describe our common effort in presenting to the international literature what we have done over the past years and indicating directions for future study. We thank in advance this prestigious journal’s Editors for offering us this opportunity.
